# Enhanced Enzyme Reuse through the Bioconjugation of L-Asparaginase and Silica-Based Supported Ionic Liquid-like Phase Materials

**DOI:** 10.3390/molecules27030929

**Published:** 2022-01-29

**Authors:** João C. F. Nunes, Mafalda R. Almeida, Rui M. F. Bento, Matheus M. Pereira, Valéria C. Santos-Ebinuma, Márcia C. Neves, Mara G. Freire, Ana P. M. Tavares

**Affiliations:** 1 CICECO-Aveiro Institute of Materials, Department of Chemistry, University of Aveiro, 3810-193 Aveiro, Portugal; jcfn@ua.pt (J.C.F.N.); mafalda.almeida@ua.pt (M.R.A.); ruib@ua.pt (R.M.F.B.); matheus.pereira@ua.pt (M.M.P.); mcneves@ua.pt (M.C.N.); maragfreire@ua.pt (M.G.F.); 2 Department of Engineering of Bioprocesses and Biotechnology, School of Pharmaceutical Sciences, São Paulo State University (UNESP), Araraquara 14800-903, Brazil; valeria.ebinuma@unesp.br

**Keywords:** L-asparaginase, silica-based supported ionic liquid-like phase materials, bioconjugation, physical adsorption, enzyme immobilization, molecular docking

## Abstract

L-asparaginase (ASNase) is an amidohydrolase that can be used as a biopharmaceutical, as an agent for acrylamide reduction, and as an active molecule for L-asparagine detection. However, its free form displays some limitations, such as the enzyme’s single use and low stability. Hence, immobilization is one of the most effective tools for enzyme recovery and reuse. Silica is a promising material due to its low-cost, biological compatibility, and tunable physicochemical characteristics if properly functionalized. Ionic liquids (ILs) are designer compounds that allow the tailoring of their physicochemical properties for a given task. If properly designed, bioconjugates combine the features of the selected ILs with those of the support used, enabling the simple recovery and reuse of the enzyme. In this work, silica-based supported ionic liquid-like phase (SSILLP) materials with quaternary ammoniums and chloride as the counterion were studied as novel supports for ASNase immobilization since it has been reported that ammonium ILs have beneficial effects on enzyme stability. SSILLP materials were characterized by elemental analysis and zeta potential. The immobilization process was studied and the pH effect, enzyme/support ratio, and contact time were optimized regarding the ASNase enzymatic activity. ASNase–SSILLP bioconjugates were characterized by ATR-FTIR. The bioconjugates displayed promising potential since [Si][N_3444_]Cl, [Si][N_3666_]Cl, and [Si][N_3888_]Cl recovered more than 92% of the initial ASNase activity under the optimized immobilization conditions (pH 8, 6 × 10^−3^ mg of ASNase per mg of SSILLP material, and 60 min). The ASNase–SSILLP bioconjugates showed more enhanced enzyme reuse than reported for other materials and immobilization methods, allowing five cycles of reaction while keeping more than 75% of the initial immobilized ASNase activity. According to molecular docking studies, the main interactions established between ASNase and SSILLP materials correspond to hydrophobic interactions. Overall, it is here demonstrated that SSILLP materials are efficient supports for ASNase, paving the way for their use in the pharmaceutical and food industries.

## 1. Introduction

**Figure 1:**
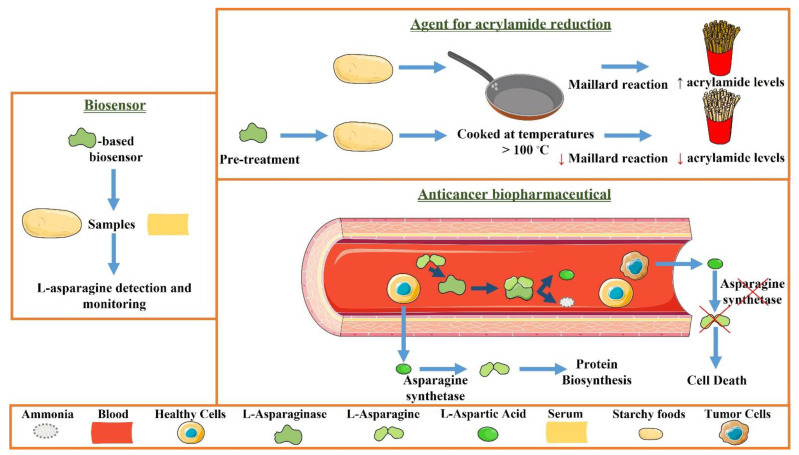
Schematic representation of the use of ASNase as an agent for acrylamide reduction in starch-rich foods cooked at temperatures over 100 °C, as an anticancer biopharmaceutical, and for L-asparagine detection and monitoring.

L-asparaginase (ASNase) (L-asparagine amidohydrolase, EC 3.5.1.1) is an enzyme able to hydrolyze L-asparagine into L-aspartic acid and ammonium. ASNase has been applied as an anticancer biopharmaceutical in the treatment of lymphoproliferative disorders, such as acute lymphoblastic leukaemia (ALL) and lymphomas, and used as an agent for acrylamide reduction in starch-rich foods cooked at high temperatures [1,2,3]. Furthermore, it has also been employed in the quantification of L-asparagine [4]. As a biopharmaceutical, ASNase acts in leukemic cells. Whereas healthy cells are able to synthesize enough L-asparagine for their needs, tumor cells are unable to synthesize this amino acid due to the lack of asparagine synthetase, and they require the free exogenous L-asparagine (Figure 1, Anticancer biopharmaceutical) [1,2,3]. Thus, tumor cells are selectively killed by L-asparagine depletion by ASNase [1,2,3]. On the other hand, in starchy foods, ASNase acts as an acrylamide reduction agent. The pre-treatment with ASNase converts L-asparagine into L-aspartic acid, decreasing the acrylamide formation through the Maillard reaction at temperatures over 100 °C (Figure 1, Agent for acrylamide reduction) [1,2,3]. Moreover, ASNase-based quantification devices have also been increasingly developed for L-asparagine detection and monitoring in pharmaceutical and food applications (Figure 1) [4].

Despite their relevance in biocatalysis, free enzymes such as ASNase display limitations, namely their single use and low stability [1,5]. This problem can be overcome by the immobilization of enzymes onto solid supports, producing a heterogeneous biocatalyst. A suitable enzyme’s immobilization on materials enables its continuous use and reuse, minimizes or eliminates product contamination, leads to high productivity while maintaining structural integrity, and may lead to enhanced catalytic activity [1,6,7]. However, previous works have reported that, depending on the enzyme immobilization method and material, the enzyme may lose its activity and reuse capacity [8]. Thus, when an immobilized biocatalyst is designed, the properties of the support and immobilization conditions must be properly and carefully considered. A suitable support has to present good biocompatibility, high physical and chemical stability, presence of multiple enzyme-support binding points, and low-cost [9], allowing, through physical adsorption, covalent attachment, or, through entrapment, the formation of the bioconjugate [1,10,11]. Physical adsorption is a low-cost method that may allow the regeneration of the support, easy reload, and enhanced performance of the enzyme [1]. On the other hand, covalent attachment is characterized by an irreversible chemical bond between the functional groups of the enzyme and the support [1]. Nevertheless, if the enzyme is immobilized in a specific orientation that makes its active sites unavailable, an enzymatic activity loss may occur [12]. Entrapment allows the preservation of the enzyme within a 3-D polymer support or the framework of a membrane [1]; however, mass transfer limitations can occur [13]. Different supports have been reported for ASNase bioconjugation, such as maltose-functionalized magnetic core/shell Fe_3_O_4_@Au nanoparticles (NPs) [14] and pristine and functionalized multi-walled carbon nanotubes (MWCNTs) [10,15]. Through covalent attachment, functionalized aluminum oxide and titanium oxide NPs [16] and magnetic Fe_3_O_4_-chitosan NPs [17] have been used, and, thorough entrapment, bovine serum albumin/ASNase/poloxamer 407 (BSA/ASNase/Pol_407_) NPs have been investigated [18].

Among all the materials used for enzymes immobilization, silica is a promising support due to its low-cost, biological compatibility, and tunable physicochemical characteristics, such as tailorable pore diameters, which allow a high surface to volume ratio and possible surface functionalization [1,19]. Hence, various enzymes, e.g., α-amylase [20], catalase [21], cytochrome c [21], glutaminase [22], invertase [23], lipase [23,24], lysozyme [21], peroxidase [21,25], protease [21], and proteinase K [25], have been successfully immobilized onto silica supports. ASNase has been investigated as well, but it is covalently confined onto silica NPs using cross-linking agents, namely 1-ethyl-3-(3-dimethylaminopropyl)carbodiimide hydrochloride (EDC) and glutaraldehyde [19]. However, glutaraldehyde can potentially be cytotoxic [26]. To improve the properties of silica, its surface can be modified by the covalent attachment of several functional groups, e.g., amine, thiol, and sulfonic groups [27]. In particular, tertiary amines have been evaluated as material’s surface modifier. Dobrzanska et al. [28] have reported that tertiary amine-functionalized materials adsorbed multilayers of aggregated lysozyme, showing that oxidation of tertiary amine decorated surfaces holds potential for selective protein deposition. Lai et al. [29] proved that ammonium ILs can offer potential advantages in facilitating enzyme functioning and that IL cations may play a main role over their counter-anions in affecting the enzyme behavior. In addition, Capela et al. [30] demonstrated that quaternary alkyl ammoniums with short alkyl side chains have beneficial effects on the stability of laccase [30], while Attri et al. [31] showed that triethyl ammonium cations with small alkyl chains of ILs (triethyl ammonium acetate and triethyl ammonium phosphate) are strong stabilizers for α-chymotrypsin.

Recently, ionic liquids (ILs) were proposed as potential surface modifying compounds for several materials, including silica [32], with potential application for the immobilization of enzymes, such as lipase [33] and penicillin G acylase [34], as well as additives for the enzyme immobilization, e.g., lipase [35], pepsin [36], and trypsin [36], through entrapment in silica matrices using sol-gel methods. ILs are composed of an organic cation and an organic or inorganic anion, thus displaying unique features if properly designed, e.g., exceptional chemical, thermal, and electrochemical stability, solvation capability, low/negligible volatility, nonflammability, among others [5,37,38]. Furthermore, ILs are considered to be designer compounds due to the high amount of possible ion combinations, allowing the tailoring of their physicochemical properties for a given task [5,37,38]. Therefore, novel materials, such as supported ionic liquid materials, which have the IL moieties covalently attached and avoid their leaching, combine the features of the selected ILs with those of the support used. If properly designed, these bioconjugates (enzymes + support material) enable the simple recovery and reusability of the enzyme, as well as the application in a fixed-bed technology for the pharmaceutical and food industries [38].

Based on these previous results from the literature, and the relevant applications of ASNase, in this work, SSILLP materials were characterized and synthesized using tertiary amines with different alkyl chain lengths as the cation source and Cl^−^ as the anion source. Different adsorption conditions were studied to improve the activity of the immobilized ASNase. At the optimal conditions, the operational stability of the bioconjugate was evaluated. A molecular docking analysis was carried out to identify the interactions between the SSILLP materials and the ASNase surface responsible for the improved enzyme activity.

## 2. Materials and Methods

### 2.1. Reagents

3-chloropropyltrimethoxysilane (≥98.0% pure), L-asparagine (99.0% pure), and tributylamine (99.0% pure) were acquired from Acros Organics—Thermo Fisher Scientific (Bridgewater, NJ, USA). Tris(hydroxymethyl) aminomethane (Tris) (99% pure) was purchased from Alfa Aesar—Thermo Fisher Scientific (Kandel, Germany). Sulfosalicylic acid (99.5%) was obtained from Carlo Erba Reagents (Val-de-Reuil, France). Ethanol (≥99.8% pure) and trioctylamine (≥98% pure) were acquired from Honeywell Research Chemicals—Inc. Fluka (Charlotte, NC, USA). Citric acid monohydrate (C_6_H_8_O_7_·H_2_O) (≥99.5% pure) was purchased from PanReac AppliChem ITW Reagents (Darmstadt, Germany). Commercial ASNase (ENZ-287) (>96.0% pure) from *Escherichia coli* ASI.357 was obtained from ProSpec-Tany TechnoGene Ltd. (Ness-Ziona, Israel). Disodium phosphate heptahydrate (Na_2_HPO_4_·7H_2_O) (≥98.0% pure), hydrochloric acid (HCl) (37.0% *w*/*w*), Nessler’s reagent (pure), *N*,*N*-dimethylbutylamine (≥98.5% pure), *N*,*N*-dimethylhexylamine (≥97.5% pure), *N*,*N*-dimethyloctylamine (≥94.0% pure), and trihexylamine (≥95.5% pure) were acquired from Sigma-Aldrich-Merck KgaA (St. Louis, MI, USA). Methanol (≥99.9% pure), toluene (≥99.8% pure), and triethylamine (≥99.0% pure) were purchased from Thermo Fisher Scientific (Waltham, MA, USA).

### 2.2. Synthesis of Silica-Based Supported Ionic Liquid-Like Phase (SSILLP) Materials

Silica was functionalized with several quaternary ammonium cations and chloride as the counterion, resulting in silica functionalized with the following functional groups: *N*,*N*-dimethylbutylammonium chloride ([Si][N_3114_]Cl), *N*,*N*-dimethylhexylammonium chloride ([Si][N_3116_]Cl), *N*,*N*-dimethyloctylammonium chloride ([Si][N_3118_]Cl), triethylammonium chloride ([Si][N_3222_]Cl), tributylammonium chloride ([Si][N_3444_]Cl), trihexylammonium chloride ([Si][N_3666_]Cl), and trioctylammonium chloride ([Si][N_3888_]Cl). The abbreviations and chemical structures of the SSILLP materials investigated are given in Table 1.

The used protocol for the synthesis of SSILLP materials was adapted from the one described by Qiu et al. [32], and previously proposed by our research group [39]. SSILLP materials’ synthesis was performed through two main steps: (i) silica activation and (ii) silica functionalization. Commercial silica gel 60 (0.2–0.5 mm) was activated through an acidic treatment with HCl (37%*w*/*w*) for 24 h. After this period, silica was washed with distilled water and dried at 60 °C for 24 h. For silica functionalization, 5 g of the activated silica were dispersed in 60 mL of toluene, placed in a round bottom flask with a reflux condenser, and 5 mL of 3-chloropropyltrimethoxysilane (C_6_H_15_ClO_3_Si) were added. The suspension was kept under reflux and magnetic stirring for 24 h. Afterwards, the resulting solid was filtrated and washed with solvents in the following order: 100 mL of toluene, 200 mL of ethanol:water 1:1 (*v*/*v*), 500 mL of distilled water, and 100 mL of methanol, and allowed to dry in an oven at 60 °C for 24 h, resulting in silica with a chloropropyl spacer arm ([Si][C_3_]Cl). During the second reaction of the silica functionalization (cation source addition), 5 g of [Si][C_3_]Cl, 60 mL of toluene, and 5 mL of a cation source, e.g., *N*,*N*-dimethylbutylamine, were put in a round bottom flask and refluxed under magnetic stirring for 24 h. Subsequently, the material was filtrated and washed with the following solvents sequence: 100 mL of toluene, 350 mL of methanol, 300 mL of distilled water, and 150 mL of methanol, and allowed to dry at 60 °C for 24 h.

### 2.3. Characterization of SSILLP Materials and Bioconjugates

All prepared SSILLP materials ([Si][N_3114_]Cl, [Si][N_3116_]Cl, [Si][N_3118_]Cl, [Si][N_3222_]Cl, [Si][N_3444_]Cl, [Si][N_3666_]Cl, and [Si][N_3888_]Cl) were characterized by elemental analysis and point of zero charge (PZC) as described below. SSILLP materials with quaternary ammonium with the shortest, intermediate, and longest alkyl chain lengths, i.e., [N_3114_], [N_3222_], and [N_3888_] and respective ASNase–SSILLP bioconjugates, i.e., ASNase-[Si][N_3114_]Cl, ASNase-[Si][N_3222_]Cl, and ASNase-[Si][N_3888_]Cl, were also characterized by attenuated total reflectance-Fourier-transform infrared spectroscopy (ATR-FTIR) studies.

#### 2.3.1. Elemental Analysis

The weight percentages (%*w*/*w*) of carbon (%C), hydrogen (%H), and nitrogen (%N) of [Si][C_3_]Cl and all SSILLP materials were determined by elemental analysis using the equipment TruSpec 630-200-200, 2 mg of sample, combustion furnace temperature of 1075 °C, and subsequent burner temperature of 850 °C. The detection method for carbon and hydrogen was infrared absorption and for nitrogen was thermal conductivity.

The bonding amount (BA) (µmol m^−2^) of [Si][C_3_]Cl was calculated taking into account the amount of carbon, according to (Equation (1)):

(1) $$\mathrm{BA}=\frac{\frac{\%C}{3\times M\left(C\right)}}{S_{\mathrm{BET}}}$$

where %C is the weight percentage of carbon and M(C) is the molar mass of carbon (g µmol^−1^). S_BET_ is the specific surface area of the initial silica determined by N_2_ isotherm adsorption measurements and Brunauer–Emmett–Teller (BET) theory, previously reported by our research group (435 m^2^ g^−1^) [39].

The BA (µmol m^−2^) of ILs onto all SSILLP materials was calculated taking into account the amount of nitrogen present, according to Equation (2):

(2) $$\mathrm{BA}=\frac{\frac{\%N}{1\times M\left(N\right)}}{S_{\mathrm{BET}}}$$

where %N is the weight percentage of nitrogen, M(N) is the molar mass of nitrogen (g µmol^−1^), and S_BET_ is the specific surface area of the initial silica (435 m^2^ g^−1^).

#### 2.3.2. Point of Zero Charge

The PZC of silica, [Si][C_3_]Cl, and all SSILLP materials were acquired through zeta potential measurements of aqueous suspensions of the materials in a wide range of pH values. To adjust the pH of the materials’ suspensions, aqueous solutions of NaOH and HCl at 0.01 M were used. Analyses were performed using a Malvern Zetasizer Nano ZS (Malvern Instruments Ltd., Malvern, UK) at 25 °C.

#### 2.3.3. Attenuated Total Reflectance-Fourier-Transform Infrared (ATR-FTIR) Spectroscopy

ATR-FTIR was performed using Perkin Elmer FT-IR System Spectrum BX equipment (Waltham, MA, USA) and a solid sample of the SSILLP materials modified with quaternary ammonium with the shortest, intermediate, and longest alkyl chain length, and respective ASNase–SSILLP bioconjugates (immobilization conditions: pH 8, 6 × 10^−3^ mg of ASNase per mg of SSILLP material, and 60 min of contact time) at 25 °C and in a range between 4000–500 cm^−1^. The samples were scanned 64 times with a resolution of 8.0 cm^−1^.

### 2.4. L-Asparaginase (ASNase) Immobilization Conditions

The ASNase immobilization was performed evaluating the pH, enzyme/support ratio, and immobilization contact time through the addition of 1 mL of an ASNase solution (0.08 mg mL^−1^, unless otherwise stated) to 10 mg of the SSILLP materials. The solution was further mixed in a programmable rotator Multi Bio RS-24 with PRS-26 platform (BioSan SIA, Riga, Latvia) (Orbital rotation 50/1, reciprocal rotation 5/1, vibro 5/1) for 60 min at 25 °C. Room temperature was selected in order to develop a low-cost process of ASNase bioconjugation, as previously applied by our research group in ASNase immobilization [10,15]. To assess the effect of pH in ASNase immobilization, ASNase solution was prepared by dissolving commercial ASNase in buffers at different pH values as follows: pH 5 and pH 6 were prepared by mixing 0.1 M citric acid solution and 0.2 M disodium hydrogen phosphate solution in different volume ratios; pH 7 and pH 8 were prepared by mixing 0.2 M disodium hydrogen phosphate solution and 0.2 M monosodium phosphate solution in different volume ratios. The selected pH range (pH 5–8) was based on previous studies from our research group regarding ASNase immobilization [10,15]. The optimal enzyme/support ratio was evaluated by immobilizing ASNase at different enzyme concentrations 0.01, 0.02, 0.03, 0.04, 0.06, and 0.08 mg mL^−1^, which correspond to an enzyme/support ratio (ASNase/SSILLP material ratio) of 1, 2, 3, 4, 6, and 8 × 10^−3^ mg of ASNase per mg of SSILLP, respectively, and an activity of 1.6 U mL^−1^, 3.1 U mL^−1^, 4.7 U mL^−1^, 6.3 U mL^−1^, 9.4 U mL^−1^, and 12.5 U mL^−1^, respectively, at pH 8, 60 min of contact time. The contact time was assessed using previously optimized conditions (pH 8 and 6 × 10^−3^ mg of ASNase per mg of SSILLP material) in different intervals of time: 30, 45, 60, 90, and 120 min. Following the immobilization tests, the mixture was centrifuged at 9600× *g* for 10 min, the supernatants were recovered, and the SSILLP materials were collected. All immobilization tests were carried out in triplicate.

The immobilized activity yield of ASNase (η_IA_) (%) onto the materials was determined according to Equation (3):

(3) $$\eta_{\mathrm{IA}}=\frac{A_{\mathrm{FA}}-A_{S}}{A_{\mathrm{FA}}}\times 100$$

where A_FA_ is the activity of free ASNase (U mL^−1^) and A_S_ is the ASNase activity in the supernatant (U mL^−1^).

### 2.5. ASNase Activity

The used protocol is an adaptation of the protocol described by Magri et al. [40]. To determine the free enzyme activity, 50 μL of ASNase solution, 450 μL of distilled water, 500 μL of Tris-HCl 50 mM pH 8.6, and 50 μL of L-asparagine 189 mM were mixed. After incubation at 37 °C for 30 min, previously defined as the optimal incubation time by our research group [10,15], the enzymatic reaction was stopped by the addition of 250 μL of sulfosalicylic acid solution 1.5 M. The ammonium produced in the L-asparagine hydrolysis by ASNase, and directly proportional to the ASNase activity, was quantified by the Nessler method. For this, 100 µL of the stopped reaction sample was added to 2.15 mL of water and 250 µL of Nessler’s reagent and incubated for 30 min at room temperature. Then, the sample’s absorbance was determined at 436 nm using a multimode microplate reader Synergy HT (BioTek, Winooski, VE, USA). A calibration curve was previously prepared through the reaction of Nessler’s reagent with ammonium sulfate aqueous solution at different concentrations. The free enzyme activity (A_FA_) was calculated according to Equation (4), and it is expressed in µmol mL^−1^ min ^−1^ (U mL^−1^).

(4) $$A_{\mathrm{FA}}=\frac{{\mathrm{NH}}_{4}^{+}\times V_{R}\times V_{N}}{V_{S}\times R_{T}\times V_{\mathrm{ASNase}}}$$

where NH_4_^+^ is the ammonium concentration produced in the enzymatic reaction (µmol mL^−1^), V_R_ is the volume of enzymatic reaction (1.05 mL), V_N_ is the volume of the Nessler reaction (2.5 mL), V_S_ is the volume of the stopped reaction sample (0.1 mL), R_T_ is the reaction time (30 min), and V_ASNase_ is the volume of ASNase (0.05 mL). One unit of ASNase activity (U) corresponds to 1 µmol of NH_4_^+^ produced per minute at pH 8.6 and 37 °C.

The activity of immobilized ASNase (A_IA_) was quantified in a reaction system containing 450 μL distilled water, 500 μL Tris-HCl 50 mM pH 8.6, 50 μL L-asparagine 189 mM, and 10 mg of the bioconjugate ASNase-SSILLP. After incubation at 37 °C for 30 min, the enzymatic reaction was stopped by the addition of 250 μL of sulfosalicylic acid solution 1.5 M. The ammonium produced in the L-asparagine hydrolysis by ASNase, and directly proportional to the ASNase activity, was quantified by the Nessler method in the same conditions as previously detailed. The immobilized enzyme activity (A_IA_) was calculated according to Equation (5), and it is expressed in µmol mg^−1^ min ^−1^ (U mg^−1^).

(5) $$A_{\mathrm{IA}}=\frac{{\mathrm{NH}}_{4}^{+}\times V_{R}\times V_{N}}{V_{S}\times R_{T}\times M_{M}}$$

where NH_4_^+^ is the ammonium concentration produced in the enzymatic reaction (µmol mL^−1^), V_R_ is the volume of enzymatic reaction (1 mL), V_N_ is the volume of the Nessler reaction (2.5 mL), V_S_ is the volume of the stopped reaction sample (0.1 mL), R_T_ is the reaction time (30 min), and M_M_ is the mass of the SSILLP materials (10 mg). One unit of ASNase activity (U) corresponds to 1 µmol of NH_4_^+^ produced per minute at pH 8.6 and 37 °C.

Control experiments were performed to evaluate possible interference of the material on the enzymatic reaction conditions. The materials were mixed with buffer solutions without ASNase at the same experimental conditions. The immobilization, enzymatic reaction, and ammonium quantification were performed as described above and no reaction was detected.

### 2.6. Operational Stability

Under the optimized immobilization conditions (pH 8, 6 × 10^−3^ mg of ASNase per mg of SSILLP material, and 60 min of immobilization contact time), after the first cycle of reaction between immobilized ASNase on the synthesized SSILLP materials ([Si][N_3114_]Cl, [Si][N_3116_]Cl, [Si][N_3118_]Cl, [Si][N_3222_]Cl, [Si][N_3444_]Cl, [Si][N_3666_]Cl, and [Si][N_3888_]Cl) and L-asparagine, the supernatants were removed and the remaining enzyme activity measured, while the immobilized ASNase was washed for 1 min with 1 mL of phosphate buffer pH 8. Afterwards, a new cycle of reaction was performed following the same protocol as described above. The relative activity of immobilized ASNase (RA_IA_) (%) was calculated according to Equation (6):

(6) $${\mathrm{RA}}_{\mathrm{IA}}=\frac{A_{\mathrm{IAC}}}{A_{\mathrm{IAC}1}}\times 100$$

where A_IAC_ is the activity of immobilized ASNase (U mg^−1^) of the SSILLP in each cycle, and A_IAC1_ is the activity of immobilized ASNase of the corresponding SSILLP in cycle 1 (U mg^−1^).

### 2.7. Molecular Docking

ASNase protonotation states of titratable residues were calculated using ProteinPrepare (PlayMolecule web server—playmolecule.org, accessed on 20 May 2021) [41]. ASNase PDB file (PDB: 2p2d) was downloaded from Protein Data bank and uploaded in ProteinPrepare application. The pKa calculation was performed at pH values from 5 to 8 without water molecules and ligands from input PDB file. After calculation, the protonated PDB files and protonation tables were downloaded and analyzed. Electrostatic properties were calculated using automatically configured sequential focusing multigrid calculation on Adaptive Poisson–Boltzmann Solver (APBS).

The interaction of ASNase with the ligands (ILs cations from SSILLP materials) was identified using the Auto-dock vina 1.1.2 program [42]. The crystal structure of ASNase (PDB: 2p2d) was prepared as described at pH 8 and applied for molecular docking. Auto DockTools (ADT) [43] was used to prepare the ASNase input file. Ligands 3D atomic coordinates were created using Discovery Studio, v20 (Accelrys, San Diego, CA, USA), applied to Chem3D-MM2 protocol for energy minimization [44], and ligand rigid root was generated using ADT. The grid center at the center of mass of ASNase was 22.198 × 23.08 × 59.903 in the *x*-, *y*-, and *z*-axes, respectively. The grid dimension was 40 × 60 × 40. The binding model that has the lowest binding free energy was searched out from 10 different conformers for each ligand. The complex of ASNase and SSILLP cations was visualized and analyzed using Discovery Studio, v20 (Accelrys, San Diego, CA, USA).

## 3. Results and Discussion

### 3.1. Characterization of SSILLP Materials

An elemental analysis was performed to quantify the content of carbon, hydrogen, and nitrogen of the synthesized SSILLP materials by covalent attachment, whose results are depicted in Table 2. All the SSILLP materials contain carbon, hydrogen, and nitrogen, whose weight percentages range from 5.74 to 10.92%, 1.34 to 2.33%, and 0.07 to 0.99%, respectively, proving the successful covalent attachment of ILs to the silica surface. These results are in contrast with [Si][C_3_]Cl, which does not contain nitrogen, supporting the lack of IL organic moieties corresponding to ammonium cations. The weight percentages of carbon, hydrogen, and nitrogen, as well as the bonding amounts of ILs onto the materials for [Si][N_3114_]Cl, [Si][N_3444_]Cl, and [Si][N_3888_]Cl, are similar to those reported by Bernardo et al. [39]. Since a common volume of all the amines was employed in the synthesis, it results in SSILLP materials with distinct functionalization degrees (based on weight percentages of nitrogen). In fact, the lower molar amounts used in the synthesis (0.021, 0.014, and 0.011 mol) correspond to the amines tributylamine, trihexylamine, and trioctylamine, which results in the SSILLP materials ([Si][N_3444_]Cl, [Si][N_3666_]Cl, and [Si][N_3888_]Cl) with lower functionalization degrees (weight percentages of nitrogen of 0.15%, 0.09%, and 0.07%, respectively) and lower bonding IL amounts (0.24, 0.14, and 0.12 µmol m^−2^, respectively). Nevertheless, it should be remarked that the molar amount of the amines used did not always correspond to the same functionalization degree and bonding amount, i.e., 0.035 and 0.036 mol of amines were used in the synthesis of [Si][N_3114_]Cl and [Si][N_3222_]Cl; however, the weight percentages of nitrogen (0.77% and 0.26%) and bonding amounts (1.26 and 0.43 µmol m^−2^, respectively) are distinct. These results reinforce that not all amines react to the same extent with the total amount of [Si][C_3_]Cl present on the silica surface.

Zeta potential measurements were carried out to determine the surface charge of the SSILLP materials. Data of the zeta potential as a function of the pH for silica, [Si][C_3_]Cl, and all the synthesized SSILLP materials are given in the Supplementary Materials (Figure S1). From these data, the point of zero charge (PZC) values, defined as the pH value at which the solid particle in suspension exhibits zero net electrical charge on its surface, were determined. The respective results are given in Table 2. All the SSILLP materials display higher PZC values, ranging from 5.5 to 10.1, than both starting silica (3.4) and [Si][C_3_]Cl (4.2), supporting the presence of a cation on the surface of the SSILLP materials and the successful silica functionalization. The PZC values of the SSILLP materials increase in the following order: [Si][N_3888_]Cl < [Si][N_3666_]Cl < [Si][N_3444_]Cl < [Si][N_3222_]Cl < [Si][N_3114_]Cl < [Si][N_3116_]Cl < [Si][N_3118_]Cl, while their bonding amount increases in the following order: [Si][N_3888_]Cl < [Si][N_3666_]Cl < [Si][N_3444_]Cl < [Si][N_3222_]Cl < [Si][N_3114_]Cl < [Si][N_3118_]Cl < [Si][N_3116_]Cl. The different PZC values are a result of the degree of functionalization of the materials since the IL moieties strongly affect their surface charge, and for which the structure and charge density of the cation should also be taken into account.

**Figure 2:**
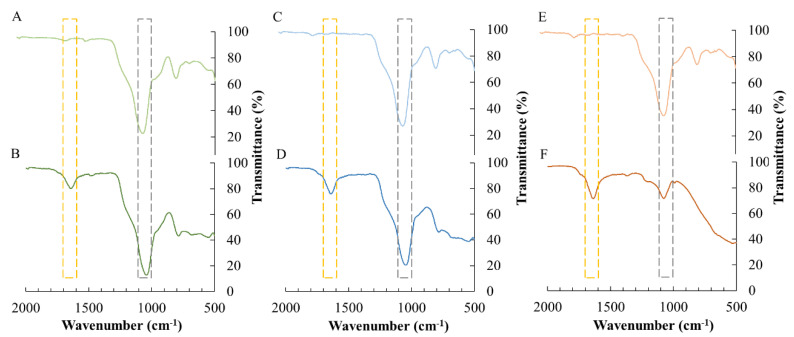
Attenuated total reflectance-Fourier-transform infrared (ATR-FTIR) spectra of [Si][N_3114_]Cl (**A**), ASNase-[Si][N_3114_]Cl (**B**), [Si][N_3222_]Cl (**C**), ASNase-[Si][N_3222_]Cl (**D**), [Si][N_3888_]Cl (**E**), and ASNase-[Si][N_3888_]Cl (**F**). 1700–1600 cm^−1^: amide group of the polypeptide chain of ASNase (dashed yellow line); 1100–1000 cm^−1^: siloxane groups (Si–O–Si) of silica (dashed grey line).

ATR-FTIR was performed to confirm the successful bioconjugation of ASNase in the SSILLP materials. The ATR-FTIR spectra (2000–500 cm^−1^) of the SSILLP materials functionalized with the quaternary ammonium with the shortest, intermediate, and longest alkyl chain lengths, i.e., [Si][N_3114_]Cl, [Si][N_3222_]Cl, and [Si][N_3888_]Cl, are shown in Figure 2A,C,E), while the ATR-FTIR spectra (2000–500 cm^−1^) of the respective ASNase–SSILLP bioconjugates, i.e., ASNase-[Si][N_3114_]Cl, ASNase-[Si][N_3222_]Cl, and ASNase-[Si][N_3888_]Cl, are given in Figure 2B,D,F). The entire ATR-FTIR spectra (4000–500 cm^−1^) (Figure S2) and spectrum (4000–500 cm^−1^) of commercial ASNase from *E. coli* used in this work (Figure S3) are depicted in the Supplementary Materials. The broad band showed in all the spectra of the materials and bioconjugates, between 1100 and 1000 cm^−1^, can be attributed to the presence of siloxane groups (Si–O–Si) of silica, as given in Figure 2 (dashed grey line) [45]. The broad band shown in the spectra of ASNase-[Si][N_3114_]Cl, ASNase-[Si][N_3222_]Cl, and ASNase-[Si][N_3888_]Cl between 1700 and 1600 cm^−1^, in contrast with the spectra of [Si][N_3114_]Cl, [Si][N_3222_]Cl, and [Si][N_3888_]Cl, is attributed to the presence of ASNase (Figure 2, dashed yellow line). These results are in accordance with the ones published by Adeishvili [46] since the major component of the FTIR spectra of native ASNase II from *E. coli* is a band at 1657 cm^−1^ [46], which corresponds to the amide group of the polypeptide chain [47]. These results confirm the successful immobilization of ASNase in the studied SSILLP materials.

### 3.2. Optimization of ASNase Immobilization Conditions

**Figure 3:**
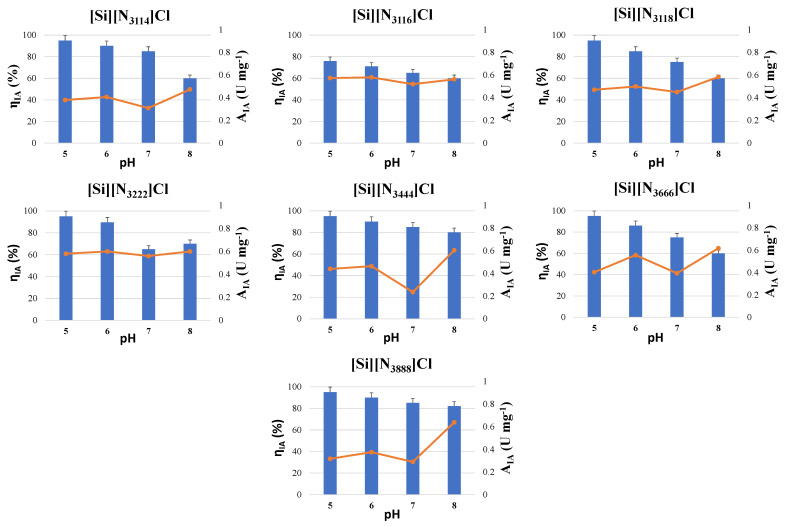
Activity of immobilized ASNase (A_IA_) (symbols, line) and immobilized activity yield (η_IA_) (bars) on the supports: [Si][N_3114_]Cl, [Si][N_3116_]Cl, [Si][N_3118_]Cl, [Si][N_3222_]Cl, [Si][N_3444_]Cl, [Si][N_3666_]Cl, and [Si][N_3888_]Cl in buffer solutions at different pH values (pH 5, 6, 7, and 8). Error bars correspond to standard deviation.

Since pH can influence the surface charge of enzymes, it is expected that this parameter will have a considerable impact regarding the ASNase immobilization onto the materials by physical adsorption. Therefore, initial tests were performed varying the pH between 5 to 8 during the immobilization of 0.08 mg mL^−1^ of ASNase onto 10 mg of all the SSILLP materials for 60 min, whose results are given in Figure 3.

The activity of the immobilized ASNase ranged from 0.32 to 0.58 U mg^−1^ at pH 5, from 0.37 to 0.6 U mg^−1^ at pH 6, from 0.24 to 0.56 U mg^−1^ at pH 7, and from 0.47 to 0.64 U mg^−1^ at pH 8 depending on the support used. The highest activities of immobilized ASNase were obtained at pH 5 (0.58 U mg^−1^), pH 6 (0.6 U mg^−1^), and pH 7 (0.56 U mg^−1^) for [Si][N_3222_]Cl; and at pH 8 for [Si][N_3222_]Cl (0.6 U mg^−1^), [Si][N_3444_]Cl (0.61 U mg^−1^), [Si][N_3666_]Cl (0.62 U mg^−1^), and [Si][N_3888_]Cl (0.64 U mg^−1^) (Figure 3). Since, for all the SSILLP materials, the highest activity of immobilized ASNase was obtained using an ASNase solution at pH 8, this value was chosen for the following assays. This optimum immobilization pH is consistent with the reported optimum pH of free ASNase [17] and in accordance with other published ASNase bioconjugation studies using other materials. In fact, the highest relative activities were obtained at pH 8 for pristine and functionalized MWCNTs [10,15] and at pH 7.5 for maltose-functionalized magnetic core/shell Fe_3_O_4_@Au NPs [14].

**Figure 4:**
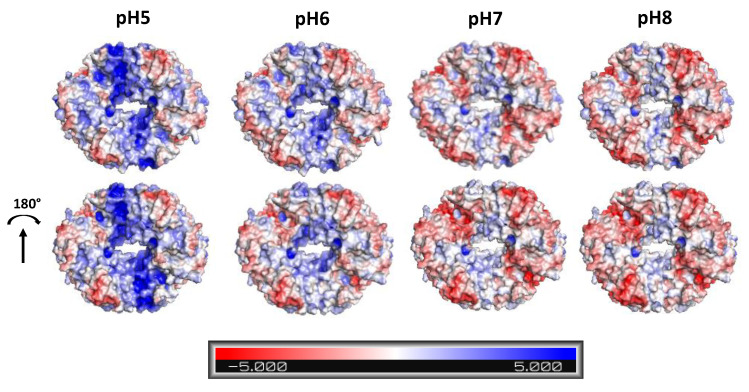
Electrostatic charge at the ASNase surface calculated for pH values from 5 to 8. Red–white–blue scale refers to minimum (-5 kT/e, red) and maximum (5 kT/e, blue) surface potential. Protein images were rendered using PyMOL (PyMOL Molecular Graphics System).

Regarding the immobilized activity yield, all the ASNase–SSILLP bioconjugates depicted the same trend since it decreases as the pH increases. The immobilized activity yields range from 76 to 95% at pH 5, from 71 to 90% at pH 6, from 65 to 85% at pH 7, and from 60 to 82% at pH 8. Computational analyses (APBS) were performed to calculate the surface charge of ASNase in the pH range from 5 to 8, with the respective protonation state being displayed in Figure 4. ASNase titratable groups identified in the enzyme tetramer are constituted by 87 amino acids (51 negatively and 36 positively charged residues). The surface of the enzyme at pH 5 presents a high number of positively charged amino acid residues (imidazolyl group from histidines, guanidinyl group from arginines, and amino group from lysines). Since the PZC values of all the SSILLP materials are higher or equal to 5.5, at pH 5, both the enzyme and the supports are positively charged, suggesting that electrostatic interactions are not responsible for the enzyme immobilization. Then, at pH 6, the reduction in the blue area (positive charge) is promoted by a transition through the ASNase isoelectric point (pI) (between 5.0 and 5.7) [48]. Therefore, above its pI, ASNase starts to display a higher red area (negative charge) based on the contribution of carboxyl groups from glutamic and aspartic acids, phenolic group from tyrosines, and sulfhydryls from cysteines. At pH 7 and pH 8, ASNase does not show significant changes in its electrostatic surface, as identified in the lower pH, displaying a negatively charged surface. All these results suggest that electrostatic interactions are not responsible for the enzyme immobilization onto the studied supports.

To improve the ASNase activity values, the influence of the enzyme/support ratio was further assessed. While keeping the mass of the SSILLP materials at 10 mg, ASNase was immobilized through adsorption onto all the SSILLP materials at pH 8 for 60 min using six ASNase/support ratios (ranging from 1 to 8 × 10^−3^ mg of ASNase per mg of SSILLP).

**Figure 5:**
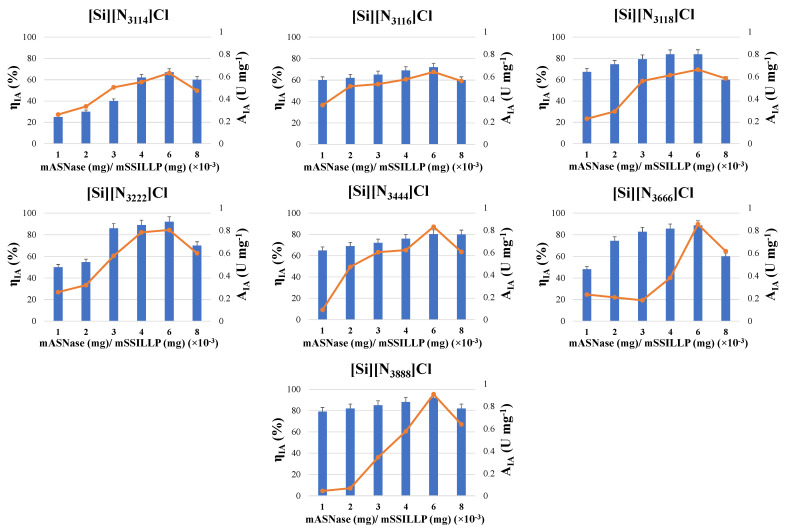
Activity of immobilized ASNase (A_IA_) (symbols, line) and immobilized activity yield (η_IA_) (bars) on the supports: [Si][N_3114_]Cl, [Si][N_3116_]Cl, [Si][N_3118_]Cl, [Si][N_3222_]Cl, [Si][N_3444_]Cl, [Si][N_3666_]Cl, and [Si][N_3888_]Cl for different enzyme/support ratio (1, 2, 3, 4, 6, and 8 × 10^−3^ mg of ASNase per mg of SSILLP). Error bars correspond to standard deviation.

The results depicted in Figure 5 show the effect of the enzyme/support ratio on the activity of immobilized ASNase. All the ASNase–SSILLP bioconjugates depicted a similar trend since the immobilized ASNase activity values increase from 1 to 6 × 10^−3^ mg of ASNase per mg of SSILLP, followed by a decrease for 8 × 10^−3^ mg of ASNase per mg of SSILLP. Since immobilized ASNase activity values up to 0.91 U mg^−1^ were obtained when using an enzyme/support ratio of 6 × 10^−3^ mg of ASNase per mg of SSILLP, this behavior is likely related to a higher amount of ASNase molecules available for the adsorption onto the surface of the SSILLP materials, forming an ASNase layer covering their surface. Nevertheless, an increase in the enzyme/support ratio up to 8 × 10^−3^ mg of ASNase per mg of SSILLP has a negative effect on the immobilized activity values, in contrast with the enzyme/support ratio of 6 × 10^−3^ mg of ASNase per mg of SSILLP, suggesting that the SSILLP materials surface achieved its maximum adsorption capacity. These results are due to mass transfer restrictions, in which it is necessary to guarantee the access of molecules of L-asparagine to the active site of ASNase [10]. Based on these results, the enzyme/support ratio of 6 × 10^−3^ mg of ASNase per mg of SSILLP was chosen for the following assays.

Concerning the immobilized activity yield, all the ASNase–SSILLP bioconjugates depicted the same trend. The immobilized activity yield increases from 25 to 92% with the increment of the enzyme/support ratio from 1 to 6 × 10^−3^ mg of ASNase per mg of SSILLP, reaching a maximum yield at 6 × 10^−3^ mg of ASNase per mg of SSILLP (immobilized activity yield of 67, 72, 83.94, 92, 80.3, 88.59, and 92% for [Si][N_3114_]Cl, [Si][N_3116_]Cl, [Si][N_3118_]Cl, [Si][N_3222_]Cl, [Si][N_3444_]Cl, [Si][N_3666_]Cl, and [Si][N_3888_]Cl, respectively), followed by a decrease at 8 × 10^−3^ mg of ASNase per mg of SSILLP.

**Figure 6:**
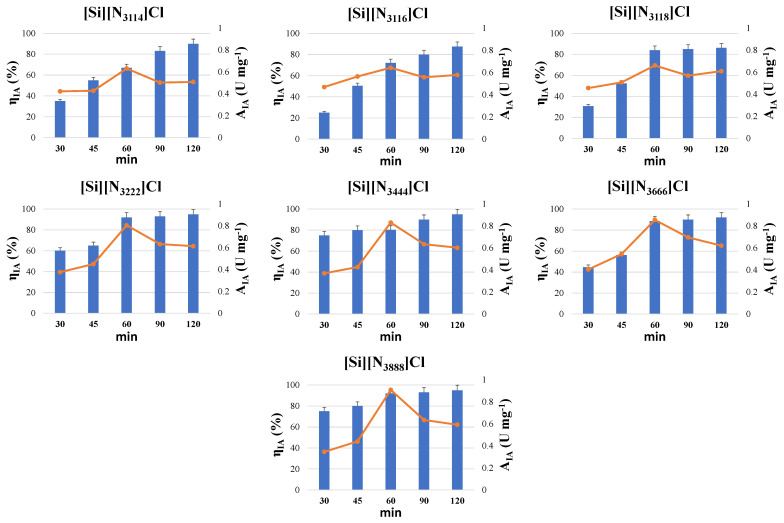
Activity of immobilized ASNase (A_IA_) (symbols, line) and immobilized activity yield (η_IA_) (bars) on the supports: [Si][N_3114_]Cl, [Si][N_3116_]Cl, [Si][N_3118_]Cl, [Si][N_3222_]Cl, [Si][N_3444_]Cl, [Si][N_3666_]Cl, and [Si][N_3888_]Cl for different ASNase immobilization contact times (30, 45, 60, 90, and 120 min). Error bars correspond to standard deviation.

To improve the ASNase immobilization conditions of the SSILLP materials, the immobilization contact time was also assessed, whose results are given in Figure 6. The immobilization of ASNase through adsorption onto all the SSILLP materials was evaluated for five contact times, between 30 and 120 min, using 6 × 10^−3^ mg of ASNase per mg of SSILLP material at pH 8. The immobilized activity values ranged from 0.35 to 0.47 U mg^−1^ for 30 min; from 0.43 to 0.56 U mg^−1^ for 45 min; from 0.63 to 0.91 U mg^−1^ for 60 min; from 0.51 to 0.70 U mg^−1^ for 90 min; and from 0.51 to 0.62 U mg^−1^ for 120 min (Figure 6). Regarding the immobilized ASNase activity, all the ASNase–SSILLP bioconjugates depicted the same trend, with the immobilized activity values increasing from 30 min up to 60 min of contact time, followed by a decrease until 120 min. These results confirm the fast ASNase immobilization onto all the SSILLP materials through a simple physical adsorption, and can be compared with the ones obtained for pristine MWCNTs, whose optimal contact time for ASNase immobilization was 45 min [10]. Since, for all the SSILLP materials, the highest activity of immobilized ASNase was obtained using 60 min of immobilization contact time, this value was chosen for the following assays, envisioning a low-cost process of ASNase bioconjugation. In fact, comparing the SSILLP materials’ performance under optimized immobilization conditions (pH 8, 6 × 10^−3^ mg of ASNase per mg of SSILLP material, and 60 min), the activity of immobilized ASNase gradually increases from 0.63 U mg^−1^ to 0.91 U mg^−1^ as the alkyl chain length of the IL cation becomes longer, supporting the relevance of hydrophobic interactions between the SSILLP materials and ASNase to improve the immobilized activity.

Regarding the immobilized activity yield, all the ASNase–SSILLP bioconjugates depicted the same trend since the immobilized activity yield increases from 25 to 95% with the increment of the contact time up to 120 min (immobilized activity yield of 90, 87.60, 86.22, 95, 95, 92, and 95% for [Si][N_3114_]Cl, [Si][N_3116_]Cl, [Si][N_3118_]Cl, [Si][N_3222_]Cl, [Si][N_3444_]Cl, [Si][N_3666_]Cl, and [Si][N_3888_]Cl, respectively). These results are similar to the ones obtained using other materials and ASNase physical adsorption. Cristóvão et al. [10] achieved a total adsorption of ASNase on pristine MWCNTs using a contact time between 90 and 120 min, and Almeida et. al. [15] reached a total adsorption of ASNase on functionalized MWCNTs using a contact time of 60 and 120 min. On the other hand, the maximum activity of immobilized ASNase achieved (0.91 U mg^−1^) was lower than reported by Morikawa et al. [49] (3.4 U mg^−1^) on ASNase-collagen membrane developed using cross-linking agents, e.g., glutaraldehyde, which can be potentially cytotoxic [26], but the maximum immobilized activity yield achieved (95%) was 1.5-fold superior (65%). Moreover, our SSILLP materials ([Si][N_3114_]Cl, [Si][N_3444_]Cl, and [Si][N_3888_]Cl) were not cytotoxic towards the HepG2 cell line (contact time of 120 min), as reported by Bernardo et al. [39], thus displaying the potential of our ASNase bioconjugation method.

To compare the performance of the different bioconjugates, the relative recovered activity of all the ASNase–SSILLP bioconjugates was assessed under optimized assay conditions (pH 8, 6 × 10^−3^ mg of ASNase per mg of SSILLP material, and 60 min). The relative recovered activity of immobilized ASNase ranged from 70 to 100% as the alkyl chain length of the IL cation becomes longer (Figure S4). Hence, [Si][N_3444_]Cl, [Si][N_3666_]Cl, and [Si][N_3888_]Cl recovered more than 92% of the initial enzyme activity (9.4 U mL^−1^), corresponding to similar values compared to the ones obtained through the physical adsorption of ASNase onto pristine MWCNTs (>90%) (optimized conditions: pH 8, 1.5 mg mL^−1^, and 60 min) [10] and onto functionalized MWCNTs (>95%) (optimized conditions: pH 8, 1.5 mg mL^−1^, and 60 min) [15] (Table 3).

### 3.3. Molecular Docking

**Figure 7:**
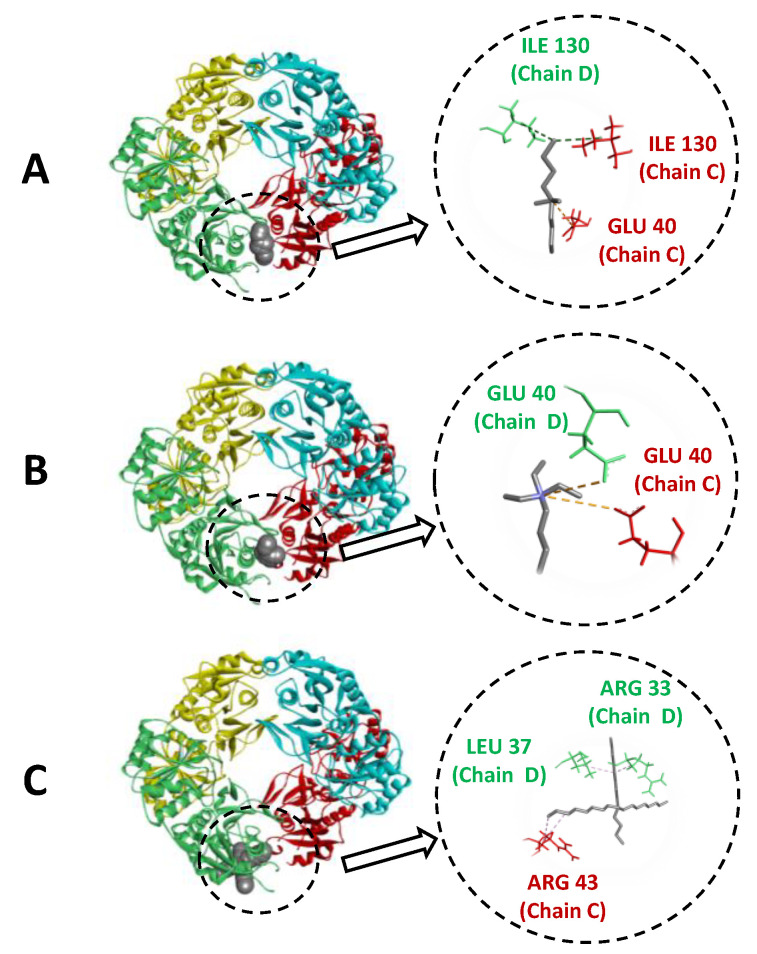
The docking pose with the lowest absolute value of affinity (kcal mol^−1^) for ASNase with SSILLP materials (cations): (**A**) [Si][N_3114_]Cl, (**B**) [Si][N_3222_]Cl, and (**C**) [Si][N_3888_]Cl. ASNase monomers are displayed in: blue (Monomer A), yellow (Monomer B), red (Monomer C), and green (Monomer D).

Aiming to identify the interactions between the SSILLP materials’ cations with the shortest, intermediate, and longest alkyl chain lengths, i.e., [N_3114_]^+^, [N_3222_]^+^, and [N_3888_]^+^ and ASNase, a molecular docking analysis was carried out. Figure 7 shows the bind pose of the IL cation with ASNase with lowest absolute value of affinity (kcal mol^−1^) with 2D diagrams of the molecular interactions. According to the docking results, the binding energy of the IL cation and ASNase follows the rank: [Si][N_3888_]Cl < [Si][N_3114_]Cl < [Si][N_3222_]Cl. However, the best immobilized enzyme activity was obtained with the SSILLP material comprising the IL longer alkyl chain length. All the SSILLP materials tested share the same Cl^−^ anion, with distinct ammonium-based cations with alkyl chain lengths containing one to eight carbons. Thus, the type of interactions occurring between the IL cation and ASNase play a critical role in terms of ruling the enzymatic performance. The type of interaction, geometry distance (Å), and interacting amino acids of all the SSILLP materials’ cations are reported in Table 4. The SSILLP materials with [N_3888_]^+^ display hydrophobic interactions with ASNase, with interactions occurring from ASNase to SSILLP materials’ cations and contrariwise. This IL with long alkyl chains promotes a higher contact with the amino acids present in the hydrophobic core of the enzyme. The SSILLP material with [N_3114_]^+^ also displays hydrophobic interactions (Table 4). However, the SSILLP materials with [N_3114_]^+^ and [N_3222_]^+^ might also interact through electrostatic interactions, derived from a higher charge density in these cations. Overall, these results suggest that the hydrophobic interactions between the IL cation and ASNase play a crucial role to improve the bioconjugate performance.

### 3.4. Operational Stability

**Figure 8:**
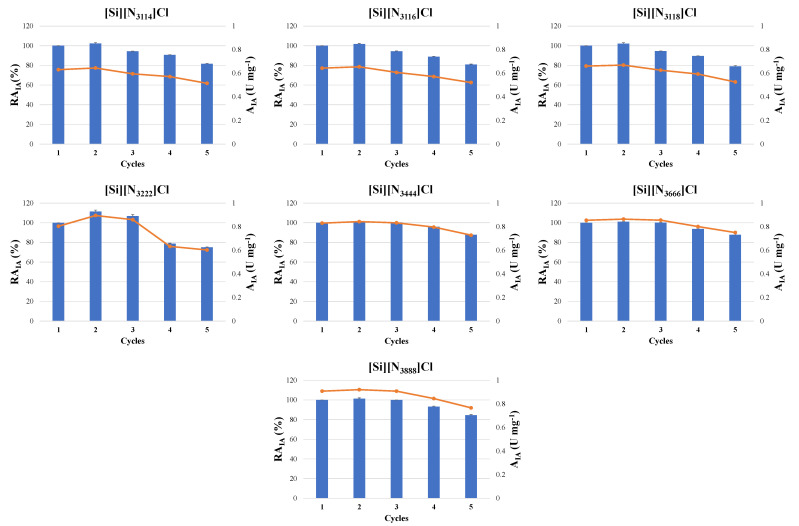
Activity of immobilized ASNase (A_IA_) (symbols, line) and relative activity of immobilized ASNase (RA_IA_) (bars) on the supports: [Si][N_3114_]Cl, [Si][N_3116_]Cl, [Si][N_3118_]Cl, [Si][N_3222_]Cl, [Si][N_3444_]Cl, [Si][N_3666_]Cl, and [Si][N_3888_]Cl during 5 cycles. Error bars correspond to standard deviation.

For a cost-effective and sustainable application, it is required to guarantee the reuse of the biocatalyst [50,51]. Therefore, the operational stability of all the ASNase–SSILLP bioconjugates was evaluated during five cycles of reaction under the previously optimized assay conditions (pH 8, 6 × 10^−3^ mg of ASNase per mg of SSILLP material, and 60 min). The respective results are presented in Figure 8.

ASNase–SSILLP bioconjugates comprising ILs with shorter alkyl chain lengths ([Si][N_3114_]Cl, [Si][N_3116_]Cl, and [Si][N_3118_]Cl) allow two cycles of reaction without losing the relative activity of immobilized ASNase, while keeping more than 79% of the initial immobilized ASNase activity after five cycles of reaction. On the other hand, ASNase–SSILLP bioconjugates with Ils with longer alkyl chain lengths ([Si][N_3222_]Cl, [Si][N_3444_]Cl, [Si][N_3666_]Cl, and [Si][N_3888_]Cl) allow three cycles of reaction without losing any relative activity of immobilized ASNase, while keeping more than 75% of the initial immobilized ASNase activity after five cycles of reaction. Therefore, these results reinforce again that hydrophobic interactions between the SSILLP materials and ASNase might be critical for maintaining the enzyme activity of the studied bioconjugates.

In order to better understand the reasons causing the decrease in the relative activity of immobilized ASNase during the five reaction cycles, the supernatants of the ASNase–SSILLP bioconjugates with the ILs composed of the longest and shortest alkyl chain lengths, i.e., [Si][N_3888_]Cl and [Si][N_3114_]Cl, were analyzed by SDS-PAGE. The results are given in the Supplementary Materials (Figure S5). Since, within the range of detection, no protein bands were obtained in any of the lanes of the supernatants of both SSILLP materials, this might mean that ASNase remains attached to the SSILLP materials’ surface throughout all the cycles of reaction. Thus, the reduction in the relative activity of immobilized ASNase might be due to the partial enzyme denaturation (unfolding of its tertiary structure to a disordered polypeptide) or to enzyme folding, while making inaccessible the active site of ASNase for L-asparagine.

Following five cycles of reaction, [Si][N_3444_]Cl, [Si][N_3666_]Cl, and [Si][N_3888_]Cl kept the initial immobilized ASNase activity between 84.4 and 88%, corresponding to similar and higher values than the ones obtained through the physical adsorption of ASNase onto maltose-functionalized magnetic core/shell Fe_3_O_4_@Au NPs (78%) [14], via covalent attachment of ASNase onto chloro-modified magnetic Fe_3_O_4_@MCM-41 core–shell NPs (81%) [51] and onto magnetic poly(HEMA-GMA) NPs (85.14%) [52], and through entrapment of ASNase onto calcium-alginate/MWCNTs-COOH (58.7%) [53] (Table 5). These results show that SSILLP materials should be deeply considered and explored as promising enzyme supports, with potential application at the industrial level.

## 4. Conclusions

SSILLP materials comprising quaternary ammoniums and chloride as counterions were successfully synthesized, characterized, and used to produce ASNase–SSILLP bioconjugates. These were produced through the simple physical adsorption of ASNase onto SSILLP materials. Several operating conditions were evaluated, and, under optimized immobilization conditions (pH 8, 6 × 10^−3^ mg of ASNase per mg of SSILLP, and 60 min), [Si][N_3444_]Cl, [Si][N_3666_]Cl, and [Si][N_3888_]Cl recovered more than 92% of the initial ASNase activity. The operational stability of the ASNase–SSILLP bioconjugates was further addressed over five cycles of reaction, being able to keep more than 75% of the initial immobilized ASNase activity. According to the experimental and molecular docking results, hydrophobic interactions are the main driving forces involved in the immobilization of ASNase over SSILLP materials and enzymatic performance. The enhanced enzymatic performance of ASNase supported onto SSILLP materials reinforces their potential to immobilize ASNase and their application for pharmaceutical and food purposes.

## Figures and Tables

**Table 1:** Ionic liquid functional group, abbreviation, and chemical structure of the SSILLP materials investigated ([Si][N_3114_]Cl, [Si][N_3116_]Cl, [Si][N_3118_]Cl, [Si][N_3222_]Cl, [Si][N_3444_]Cl, [Si][N_3666_]Cl, and [Si][N_3888_]Cl). The intermediate material [Si][C_3_]Cl is also provided.

Ionic Liquid	Abbreviation	Chemical Structure
―	[Si][C_3_]Cl	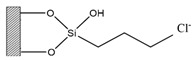
*N*,*N*-Dimethylbutylammonium chloride	[Si][N_3114_]Cl	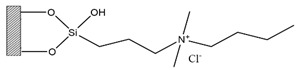
*N*,*N*-Dimethylhexylammonium chloride	[Si][N_3116_]Cl	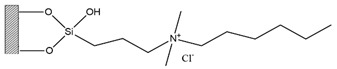
*N*,*N*-Dimethyloctylammonium chloride	[Si][N_3118_]Cl	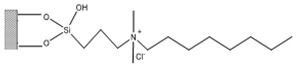
Triethylammonium chloride	[Si][N_3222_]Cl	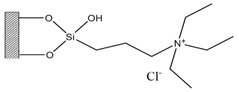
Tributylammonium chloride	[Si][N_3444_]Cl	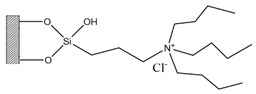
Trihexylammonium chloride	[Si][N_3666_]Cl	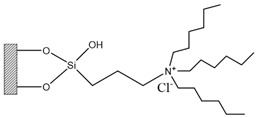
Trioctylammonium chloride	[Si][N_3888_]Cl	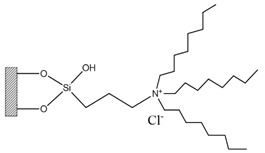

**Table 2:** Elemental analysis results: weight percentages of carbon (%C), hydrogen (%H), and nitrogen (%N); molar amount (n (mol)) of each amine used in the synthesis; bonding amount (BA) (µmol m^−2^); and point of zero charge (PZC) of [Si][C_3_]Cl and all SSILLP materials ([Si][N_3114_]Cl, [Si][N_3116_]Cl, [Si][N_3118_]Cl, [Si][N_3222_]Cl, [Si][N_3444_]Cl, [Si][N_3666_]Cl, and [Si][N_3888_]Cl).

Material	%C	%H	%N	n (mol)	BA (µmol m^−2^)	PZC
Silica	―	―	―	―	―	3.4
[Si][C_3_]Cl	4.64	1.39	0.00	―	2.96	4.2
[Si][N_3114_]Cl	7.72	1.84	0.77	0.035	1.26	9.3
[Si][N_3116_]Cl	10.72	2.32	0.99	0.028	1.62	10.0
[Si][N_3118_]Cl	10.92	2.33	0.79	0.023	1.29	10.1
[Si][N_3222_]Cl	7.29	1.51	0.26	0.036	0.43	9.0
[Si][N_3444_]Cl	5.74	1.34	0.15	0.021	0.24	6.0
[Si]N_3666_]Cl	6.55	1.52	0.09	0.014	0.14	5.9
[Si][N_3888_]Cl	6.90	1.34	0.07	0.011	0.12	5.5

**Table 3:** Comparison between this work and literature results on the immobilization of commercial ASNase from *E. coli* through a simple physical adsorption regarding support and relative recovered activity in relation to the initial free ASNase activity.

Support	Relative Recovered Activity *	Refs.
[Si][N_3444_]Cl	92%	This work
[Si][N_3666_]Cl	95%	
[Si][N_3888_]Cl	100%	
Pristine MWCNTs	>90%	[10]
Functionalized MWCNTs	>95%	[15]

* Residual ASNase activity in relation to the initial free ASNase activity (%).

**Table 4:** Docking affinity energy, type of interaction, interaction from SSILLP material cation or ASNase amino acid to ASNase amino acid or SSILLP material cation, and geometry distance (Å) for ASNase with SSILLP materials’ cations.

SSILLP Cation	Affinity (kcal mol^−1^)	Type of Interactions	Interaction From	To	Distance (Å)
[N_3222_]^+^	−5.1	Electrostatic	[N_3222_]^+^	GLU40 ^1^* (Chain C)	5.57
				GLU40 (Chain D)	5.18
[N_3114_]^+^	−5.5	Electrostatic	[N_3114_]^+^	GLU40 (Chain C)	5.02
		Hydrophobic		ILE130 ^2^* (Chain C)	4.64
				ILE130 (Chain D)	4.89
[N_3888_]^+^	−7.4	Hydrophobic	ARG43 ^3^* (Chain C)	[N_3888_]^+^	5.46
			ARG33 (Chain D)	[N_3888_]^+^	4.01
			[N_3888_]^+^	LEU37 ^4^* (Chain D)	4.74
			[N_3888_]^+^	ARG43 (Chain C)	5.16

* Amino acids: ^1^ Glutamic acid; ^2^ isoleucine; ^3^ arginine; ^4^ leucine.

**Table 5:** Comparison between this work and literature results on the immobilization of commercial ASNase from *E. coli* regarding immobilization method, support, and residual ASNase activity after 5 cycles of reaction in relation to the initial ASNase activity.

Immobilization Method	Support	Enzyme Activity after 5 Cycles of Reaction *	Refs.
Physical adsorption	[Si][N_3444_]Cl	88%	This work
	[Si][N_3666_]Cl	88%	
	[Si][N_3888_]Cl	84.4%	
	Maltose-functionalized magnetic core/shell Fe_3_O_4_@Au NPs	≈78%	[14]
Covalent attachment	Chloro-modified magnetic Fe_3_O_4_@MCM-41 core-shell NPs	≈81%	[51]
	Magnetic poly(HEMA-GMA) NPs	85.14%	[52]
Entrapment	Calcium-alginate/MWCNTs-COOH	58.7%	[53]

* Residual ASNase activity in relation to the initial immobilized ASNase activity (%).

## Data Availability

Not applicable.

## Supplementary Materials

The following supporting information can be downloaded online, Figure S1: Zeta potential (ZP) of silica, [Si][C_3_]Cl, and all SSILLP materials ([Si][N_3114_]Cl, [Si][N_3116_]Cl, [Si][N_3118_]Cl, [Si][N_3222_]Cl, [Si][N_3444_]Cl, [Si][N_3666_]Cl, and [Si][N_3888_]Cl) as a function of pH. Figure S2: Attenuated total reflectance-Fourier-transform infrared (ATR-FTIR) spectra of [Si][N_3114_]Cl (A), ASNase-[Si][N_3114_]Cl (B), [Si][N_3222_]Cl (C), ASNase-[Si][N_3222_]Cl (D), [Si][N_3888_]Cl (E), and ASNase-[Si][N_3888_]Cl (F). Figure S3: ATR-FTIR spectrum of the commercial ASNase from *E. coli* used in this work. Figure S4: Relative recovered activity of immobilized ASNase (RRA_IA_) on the supports: [Si][N_3114_]Cl, [Si][N_3116_]Cl, [Si][N_3118_]Cl, [Si][N_3222_]Cl, [Si][N_3444_]Cl, [Si][N_3666_]Cl, and [Si][N_3888_]Cl under optimized assay conditions (pH 8, 6 × 10^−3^ mg of ASNase per mg of SSILLP, and 60 min). Error bars correspond to standard deviation. Figure S5: SDS-Page of the supernatants of the assays performed in the operational stability studies (5 cycles of reaction) for the SSILLP materials [Si][N_3888_]Cl and [Si][N_3114_]Cl; lane 1: Full-Range Rainbow Molecular Weight Marker by Cytiva (protein molecular weight marker), lane 2: 0.06 mg mL^−1^ of ASNase, lanes 3, 4, 5, 6, and 7: cycles 1, 2, 3, 4, and 5 of [Si][N_3888_]Cl, lanes 8, 9, 10, 11, 12: cycles 1, 2, 3, 4, and 5 of [Si][N_3114_]Cl.

## Sample Availability

Samples of the compounds are not available from the authors.
