# Supplementary material for: Enhanced Enzyme Reuse through the Bioconjugation of L-Asparaginase and Silica-Based Supported Ionic Liquid-like Phase Materials
Source: Molecules. 2022 Jan 29;27(3):929. doi: 10.3390/molecules27030929 (PMC8838661; doi:10.3390/molecules27030929)
Supplement: Supplementary file 1 [file molecules-27-00929-s001.zip › molecules-1547126-supplementary.pdf]

Supplementary Materials

Enhanced enzyme reuse through the bioconjugation of L-asparaginase and silica-based supported ionic-liquid like phase materials

João C. F. Nunes [1], Mafalda R. Almeida [1], Rui M. F. Bento [1], Matheus M. Pereira [1], Valéria C. Santos-Ebinuma [2], Márcia C. Neves [1], Mara G. Freire [1] and Ana P. M. Tavares [1,*]

[1] CICECO-Aveiro Institute of Materials, Department of Chemistry, University of Aveiro, 3810-193 Aveiro, Portugal;

[2] Department of Engineering of Bioprocesses and Biotechnology, School of Pharmaceutical Sciences, São Paulo State University (UNESP), Araraquara 14800-903, Brazil.

---

* *Correspondence: aptavares@ua.pt (A.P.M.T.)*

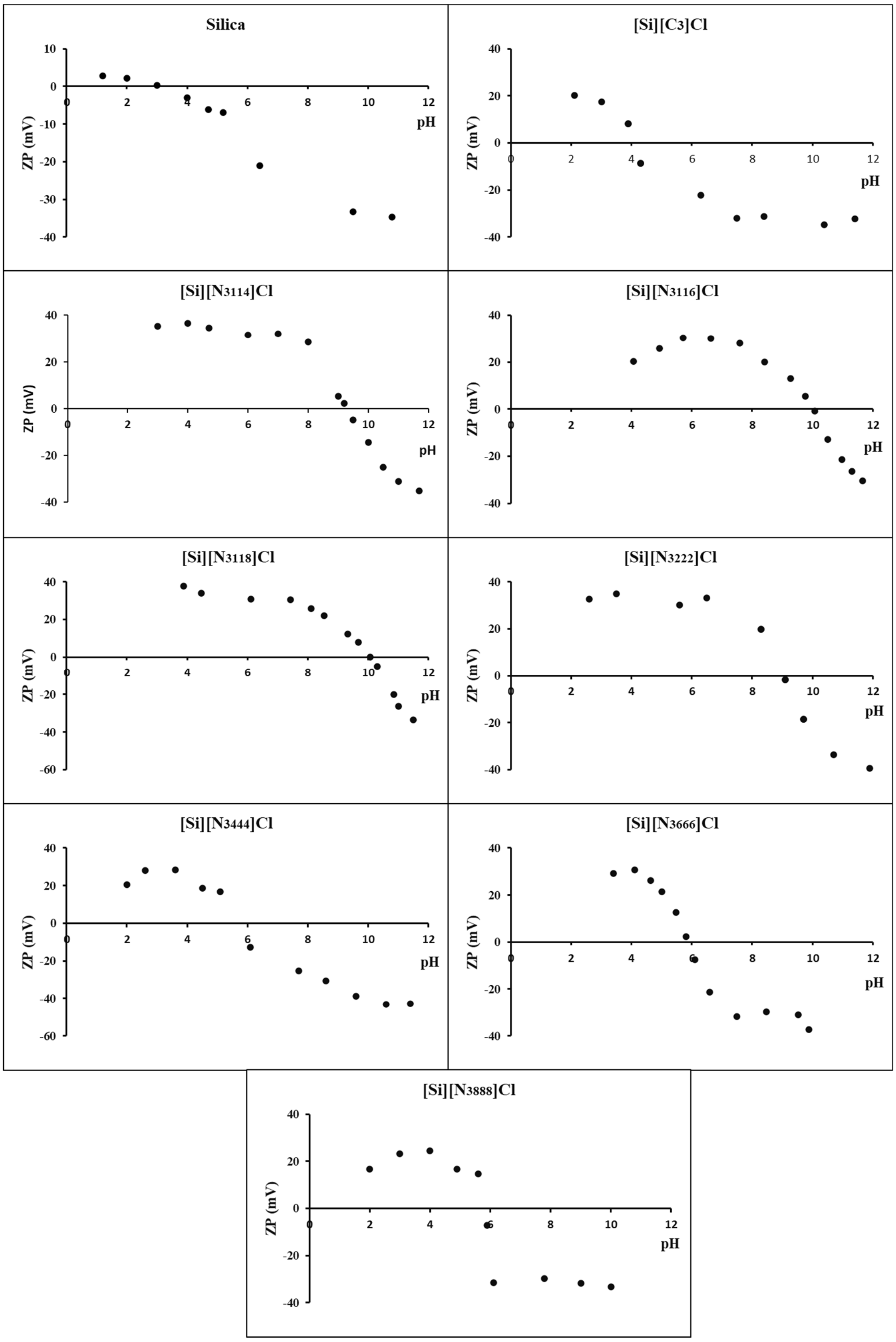

Figure S1. Zeta potential (ZP) of silica, [Si][C_3]Cl and all SSILLP materials ([Si][N_{3114}]Cl, [Si][N_{3116}]Cl, [Si][N_{3118}]Cl, [Si][N_{3222}]Cl, [Si][N_{3444}]Cl, [Si][N_{3666}]Cl and [Si][N_{3888}]Cl) as a function of pH.

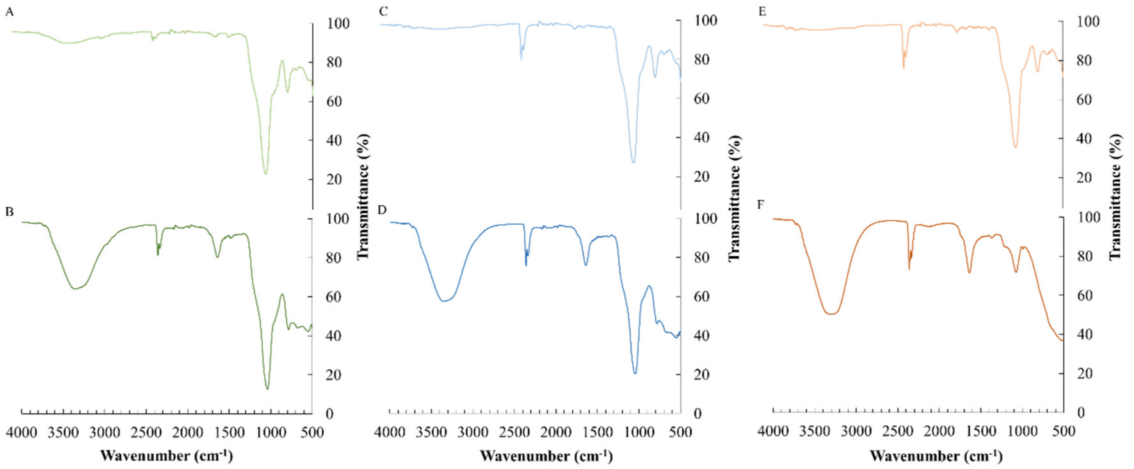

Figure S2. Attenuated total reflectance - Fourier-transform infrared (ATR-FTIR) spectra of [Si][N_{3114}]Cl (A), ASNase-[Si][N_{3114}]Cl (B), [Si][N_{3222}]Cl (C), ASNase-[Si][N_{3222}]Cl (D), [Si][N_{3888}]Cl (E), and ASNase-[Si][N_{3888}]Cl (F).

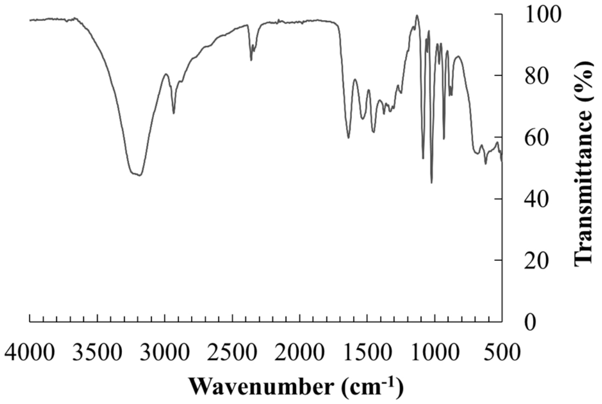

Figure S3. ATR-FTIR spectrum of the commercial ASNase from *E. coli* used in this work.

The relative recovered activity of immobilized ASNase (RRA_{IA}) (%) onto the SSILLP materials was assessed using the following conditions: constant ASNase concentration (6×10^{-2} mg mL^{-1}), volume of the Nessler reaction (V_N: 2.5 mL), volume of the stopped reaction sample (V_S: 0.1 mL), reaction time (R_T: 30 min) and was determined according to Eq. S1:

$$RRA_{IA} = \frac{A_{IA}}{MA_{FA}} \times 100 \text{ (S1)}$$

where A_{IA} (U mg^{-1}) is the activity of the effectively immobilized enzyme and MA_{FA} (U mg^{-1}) is the maximum theoretical activity that would exist if the free enzyme was totally immobilized (U mg^{-1}) determined according to Eqs. S2 and S3:

$$A_{IA} = \frac{NH_4^+ \times V_R}{M_M} \text{ (S2)}$$

$$MA_{FA} = \frac{NH_4^+ \times d_f}{M_M} \text{ (S3)}$$

where NH_4^+ is the ammonium concentration produced in the enzymatic reaction (μmol mL^{-1}), V_R is the volume of enzymatic reaction (1 mL), M_M is the mass of the SSILLP materials (10 mg) and d_f is the dilution factor.

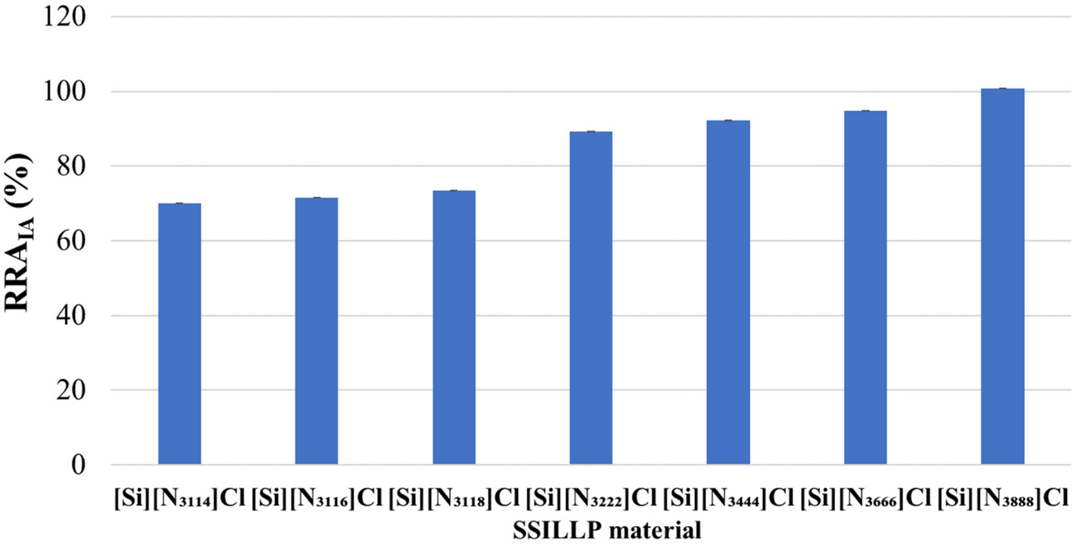

Figure S4. Relative recovered activity of immobilized ASNase (RRA_{IA}) on the supports: $[Si][N_{3114}]Cl$, $[Si][N_{3116}]Cl$, $[Si][N_{3118}]Cl$, $[Si][N_{3222}]Cl$, $[Si][N_{3444}]Cl$, $[Si][N_{3666}]Cl$ and $[Si][N_{3888}]Cl$ under optimized assay conditions (pH 8, 6×10^{-3} mg mg^{-1} of ASNase and 60 min). Error bars correspond to standard deviation.

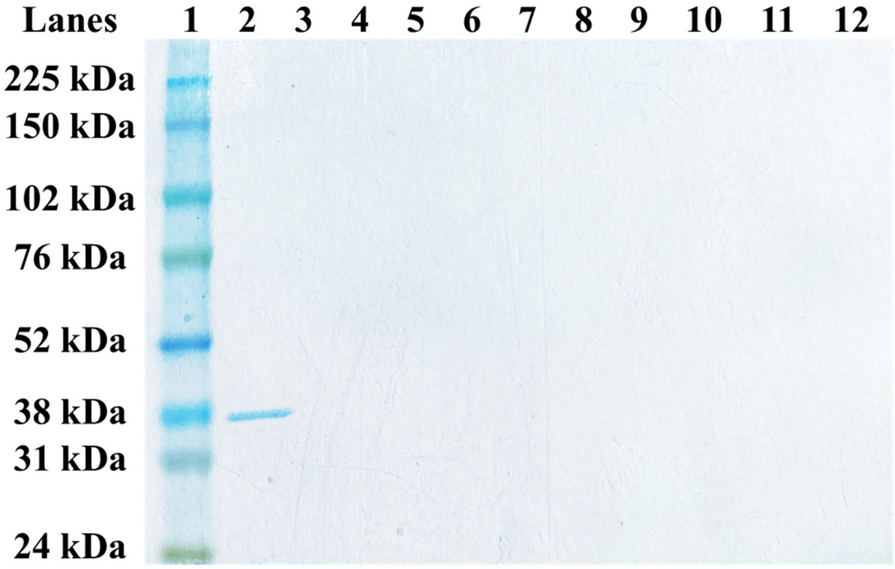

Figure S5. SDS-Page of the supernatants of the assays performed in the operational stability studies (5 cycles of reaction) for the SSILLP materials $[Si][N_{3888}]Cl$ and $[Si][N_{3114}]Cl$; lane 1: Full-Range Rainbow Molecular Weight Marker by Cytiva (protein molecular weight marker), lane 2: 0.06 mg mL^{-1} of ASNase, lanes 3, 4, 5, 6, and 7: cycles 1, 2, 3, 4, and 5 of $[Si][N_{3888}]Cl$, lanes 8, 9, 10, 11, 12: cycles 1, 2, 3, 4, and 5 of $[Si][N_{3114}]Cl$.
